# Structural heterogeneity of the psychosocial network in adolescent depression: a comparison between first-episode and recurrent patients

**DOI:** 10.3389/fpsyt.2026.1918132

**Published:** 2026-08-13

**Authors:** Ruiqiong Xiao, Gege Li, Yingjie Yao, Min Yang

**Affiliations:** Xiangya School of Nursing, Central South University, Changsha, China

**Keywords:** adolescent depression, digital compensation, network analysis, psychosocial network, recurrence, self-esteem

## Abstract

**Background:**

Adolescent depression is a recurrent disorder characterized by progressively severe psychosocial impairment, which is frequently accompanied by a fundamental structural shift in the patient’s support ecosystem from real-world interactions to virtual networks. However, the structural heterogeneity of the broader psychosocial network between first-episode and recurrent patients remains unclear.

**Methods:**

We analyzed cross-sectional data from 471 adolescents diagnosed with depression, including 171 first-episode and 300 recurrent patients. The study assessed 14 dimensions encompassing internet support, school connection, social adaptation, family care, self-esteem, and depressive symptoms. We estimated Gaussian Graphical Models and conducted a Network Comparison Test to evaluate structural differences between disease stages.

**Results:**

In the overall network, interpersonal harmony emerged as the primary central hub, while self-esteem functioned uniquely as a system stabilizer. The Network Comparison Test revealed significant structural differences between the two groups (Max Edge *M* = 0.243, *P* = 0.039). Specifically, the association between family care and learning autonomy was absent in the recurrent group (*P* < 0.01). Instead, family care formed a newly emerged, significant connection with self-esteem in recurrent patients (*P* < 0.01). Furthermore, recurrent adolescents demonstrated significantly stronger compensatory connections in the digital domain, particularly between online information support and life independence, and between online emotional support and classmate support (*P* < 0.05).

**Conclusions:**

Adolescent depression networks show clinical-stage heterogeneity. Globally, interpersonal harmony acts as the primary central hub, while self-esteem functions as a system stabilizer. In recurrent patients, real-world academic associations are absent; instead, family care forms a specific correlation with self-esteem regulation, and digital networks provide compensatory social connections. Nursing interventions should universally target interpersonal relationships and the cognitive rebalancing of self-esteem. For recurrent patients, clinicians must prioritize family-based self-esteem regulation and utilize digital communities as transitional bridges for social functioning.

## 1 Introduction

Depression has emerged as a major public health challenge among adolescents worldwide. According to the latest data from the World Health Organization (1), approximately one in seven adolescents aged 10 to 19 years suffers from a mental disorder, with depression being one of the leading causes of disease burden and disability in this age group (2, 3). In China, patients under 18 years account for 30% of all depression cases (4), and the prevalence of depression among adolescents has reached 15% to 20% (5), indicating a clear trend of earlier age of onset and increasing incidence (6). Adolescence is a critical period for psychological development and the establishment of social functioning (7). Depression during this stage is characterized not only by persistent low mood but also by significant impairment in social adaptation, often resulting in academic disruption and interpersonal difficulties. Paying close attention to psychosocial well-being of depressed adolescents is clearly urgent.

Adolescent psychological defenses and social adaptation skills do not exist in isolation. They are deeply rooted in a multidimensional psychosocial network. Real-world support systems, including family care and school connection, play a basic role in buffering negative emotions, maintaining self-esteem, and promoting social adaptation. At the same time, the digital age has fully arrived, the internet has become a core social space for depressed adolescents worldwide. They use online platforms to seek emotional resonance, information support, and social interaction (8, 9). Crucially, these real-world and online factors do not operate independently; instead, they interact in highly complex, nonlinear ways—for example, poor family support may be associated with excessive online seeking, which in turn correlates with poorer face-to-face social skills and weaker real-world support.

Traditional statistical approaches (e.g., linear regression and structural equation modeling) are often designed to estimate linear, additive effects with limited capacity for modeling simultaneous, bidirectional, or feedback-based interactions among many variables. While they can include interaction terms or non-recursive paths, they quickly become impractical as the number of interacting psychosocial nodes grows. Network analysis offers an alternative by conceptualizing mental disorders and psychosocial factors as a system of interconnected nodes (10). Although basic network models (e.g., Gaussian graphical models) still assume linear associations, they allow researchers to visualize and quantify the entire web of partial correlations without *a priori* directional constraints (11). Thus, to accurately capture the dynamic complexity described above, a methodological shift—not a wholesale rejection of classical tools, but a deliberate supplementation—is warranted toward network-based approaches.

A growing body of work supports the use of network analysis in adolescent samples. For instance, Feng et al. (12)and Huang et al. (13)utilized network analysis to identify key bridging nodes between negative life events, childhood abuse, and suicidal ideation in depressed adolescents. Similarly, Li et al. (14)explored the multisystemic factors influencing depressive symptoms using a cross-lagged network approach. However, a notable gap remains. Most existing network studies have predominantly focused on micro-level symptom-to-symptom networks or the direct impact of trauma on symptom clusters. Little is known about how the broader psychosocial support ecosystem reorganizes itself as depression becomes chronic. Specifically, few studies have systematically examined how adolescents shift between traditional real-world support and modern digital support across different clinical stages (8, 12–14). This gap is concerning given that depression has a high risk of recurrence, and recurrent episodes typically bring more severe psychosocial impairment than first-episode depression (15). Moreover, the impairment of social functioning following the illness may lead to changes in real-world interactions, prompting patients to turn to virtual networks for support or assistance; consequently, the network structures of first-episode and recurrent patients may differ significantly.

We deliberately selected six common factors representing psychosocial well-being, including internet social support, school connection, family care, self-esteem, social adaptation, and depressive symptoms. These specific factors were chosen because family and school represent the traditional offline buffering systems, while internet support constitutes the modern digital exosystem. Self-esteem and social adaptation represent the internal psychological resilience and actual real-world functional outcomes, respectively (16). There are two purposes for this study, first, researchers aim to identify the core hub nodes that maintain the stability of the psychosocial network in adolescents with depression. Second, researchers aim to explore whether a statistically significant difference exists in the overall network topology between the first-episode group and the recurrent group.

## 2 Methods

### 2.1 Design and participants

A cross-sectional design guided the current investigation. Adolescent depression patients were recruited from the inpatient departments and psychiatric outpatient clinics of two large tertiary hospitals in Changsha Hunan Province between March 2025 and October 2025. Convenience sampling was used to recruit participants who met the following criteria: (1) an established clinical diagnosis of depressive disorder verified by two independent psychiatrists according to ICD-10/DSM-5 criteria, with maintained relatively stable cognitive function, emotional regulation capacity, and behavioral performance under systematic treatment, demonstrating intact logical thinking abilities and practical communication skills; (2) aged 12~18 years; and (3) obtained parental informed consent and voluntarily participated. To ensure clinical representativeness, the enrolled adolescents were not exclusively diagnosed with depressive disorder; patients with mild-to-moderate secondary anxiety symptoms were also permitted. However, to minimize confounding severe psychopathology, participants with other comorbid psychiatric conditions were strictly excluded. Specifically, the exclusion criteria included: (1) other organic or neurological diseases; (2) a history of substance abuse or dependence; and (3) comorbid severe Axis I psychiatric disorders, such as bipolar disorder, schizophrenia spectrum disorders, autism spectrum disorders, or post-traumatic stress disorder (PTSD). Ultimately, 471 eligible adolescents with depression entered the analysis. Participants were categorized into two groups based on their clinical diagnostic history. The first-episode group was defined as patients experiencing their first lifetime major depressive episode with no prior history of depressive episodes or systematic antidepressant treatment. The recurrent group was defined as patients who had experienced at least two separate major depressive episodes, with a period of complete remission (recovery of social functioning and absence of significant symptoms) lasting at least two months between the previous and current episodes. This classification was verified by two independent psychiatrists through a review of standardized electronic medical records.

The Ethics Committee of Xiangya School of Nursing at Central South University reviewed and approved the study protocol under the ethics review number E202518. The research team strictly adhered to the Declaration of Helsinki throughout the data collection process.

### 2.2 Sample size calculation

The sample size for this study was determined based on the statistical requirements for stable network estimation. In Gaussian Graphical Models, the number of estimated parameters (i.e., edges) grows with the number of nodes. For our 14-node psychosocial network, a total of 91 possible edges (14 × 13/2) required estimation. According to the established parameter-to-subject ratio rule of thumb in multivariate analysis, a minimum ratio of 5:1 (five participants per estimated parameter) is recommended to ensure model stability and prevent overfitting. Consequently, the minimum required sample size was calculated to be 455 participants (91 × 5). The final inclusion of 471 eligible adolescents with depression successfully satisfied this statistical requirement, providing sufficient statistical power for robust network estimation and the subsequent Network Comparison Test.

### 2.3 Network node selection and measurement tools

Six independent psychological measurement scales constituted the network analysis model. Fourteen core dimensions served as the extracted network nodes. Researchers strategically selected nodes based on the psychometric properties of the instruments. Unidimensional or brief screening tools, such as the Rosenberg Self-Esteem Scale and Family APGAR, entered the network as integral total scores. Conversely, highly complex multidimensional constructs, such as social adaptation and internet support, entered the network as sub-dimensions. This hybrid structural strategy avoids the extreme topological complexity of item-level networks while maintaining sufficient micro-level clinical granularity. At the theoretical level, the ecological systems theory supports this multidimensional system integration. The resulting network comprehensively covers external environmental support and internal psychological characteristics. At the methodological level, directly utilizing item-level networks would trigger extreme topological complexity. Item-level analysis often requires a massive sample size of thousands of people for adequate statistical support. The chosen structure containing 14 dimensions perfectly matches the current sample size and guarantees the statistical robustness of the Gaussian Graphical Model. It also provides a highly interpretable macroscopic psychosocial perspective for clinical practitioners. The specific measurement tools are detailed below.

#### 2.3.1 Internet social support

Researchers utilized the Adolescent Internet Social Support Scale to assess network social support (17). This tool contains 23 items covering four dimensions. These four dimensions include information support, peer support, emotional support, and instrumental support. Participants rated each item using a 5 point scoring method. Scores range from 1 for completely disagree to 5 for completely agree. Higher total scores indicate a greater perceived level of network social support. The Cronbach alpha coefficient of this scale reached 0.926 in the current analysis.

#### 2.3.2 School connection

The School Connection Scale measured school connection levels (18). The instrument contains 10 items covering three dimensions. These dimensions encompass classmate support, teacher support, and school belonging. Participants scored the items using a 5 point Likert scale method. Scores range from 1 for completely inconsistent to 5 for completely consistent. Items 2 and 10 require reverse scoring. Higher total scores reflect a stronger sense of school connection among participants. The Cronbach alpha coefficient achieved a value of 0.820.

#### 2.3.3 Social adaptation

Evaluations of social adaptation ability relied on the Child and Adolescent Social Adaptation Questionnaire (19). This questionnaire contains 34 items spanning four core dimensions. These dimensions represent interpersonal Harmony, environment identity, life Independence, and learning autonomy. Items 12 and 26 serve as lie detector questions. Participants responded using a 5 point scoring method. Scores range from 1 for completely inconsistent to 5 for completely consistent. Higher scores prove better social adaptation levels. The Cronbach alpha coefficient of this questionnaire was 0.920.

#### 2.3.4 Family care

Family function assessments utilized the Family APGAR Index (20). The questionnaire contains 5 items. Participants rated the statements using a 3 point scoring method. Scores range from 0 for almost never to 2 for often. The total score ranges from 0 to 10 points. A total score of 7 to 10 points indicates good family function. A score of 4 to 6 points to moderate family dysfunction. A score of 0 to 3 points represents severe family dysfunction. The Cronbach alpha coefficient of this questionnaire equaled 0.860.

#### 2.3.5 Self-esteem level

The Rosenberg Self-esteem Scale captured the overall self-esteem levels of the subjects (21). The scale consists of 10 items (22). Participants rated the items using a 4-point scoring method. Scores range from 1 for strongly disagree to 4 for strongly agree. Items 3, 5, 8, 9, and 10 require reverse scoring. Higher scores represent higher levels of overall self-esteem. The Cronbach alpha coefficient of this scale was 0.949.

#### 2.3.6 Depressive symptoms

Measurements of depressive symptom severity applied the Kutcher Adolescent Depression Scale 11 item version (23). The scale contains 11 items used to evaluate core depressive manifestations (24). Participants assessed symptom frequency using a 4-point scoring method. Scores range from 0 for almost none to 3 for all the time. The total score ranges from 0 to 33 points. Higher total scores indicate more severe depressive symptoms. The Cronbach alpha coefficient of this scale reached 0.840.

### 2.4 Statistical analysis

Data analysis was conducted using SPSS 27.0 and the R software environment. For descriptive statistics, continuous variables with normal distributions were presented as means and standard deviations, while categorical variables were summarized as frequencies and percentages. Group differences in demographic and clinical variables were evaluated using independent sample *t*-tests and Chi-square tests.

The R software environment executed all advanced network estimations and comparisons (25). Prior to network estimation, all 14 dimensions scores were standardized using *Z*-score transformations. To account for the significant age difference between the two groups, age was controlled as a continuous covariate by regressing all 14 standardized nodes on age and extracting the residuals—a validated procedure in network psychometrics (26).

Gaussian Graphical Models were estimated using the graphical LASSO algorithm based on the Extended Bayesian Information Criterion (EBICglasso) with a tuning parameter of 0.5 to control false positive edges. To accurately quantify the cumulative strength of positive and negative associations of each node, Expected Influence (EI) was calculated, as it robustly accounts for both positive and negative edge weights (27). Additionally, bridge expected influence (Bridge EI) was computed using the *networktools* package to identify key nodes connecting the six predefined psychosocial communities. Compared to bridge strength, Bridge EI accounts for the direction (positive or negative) of bridging edges, providing a more accurate measure of a node’s net influence across different systems (28). All network visual presentations were completed using the *qgraph* package in R software (29).

Network stability and accuracy were evaluated using the *bootnet* package (11). The analysis performed 1,000 nonparametric bootstraps to construct 95% confidence intervals for edge weights. Furthermore, a case-dropping bootstrap approach was utilized to calculate the Correlation Stability coefficient (CS-coefficient) for Expected Influence (11). A reliable network requires a CS-coefficient greater than 0.25 and preferably above 0.5.

Finally, the *NetworkComparisonTest* package in R software was used to compare the network structures between the first-episode and recurrent groups (30). This test represents a parameter-free permutation method based on 1000 permutations. Network heterogeneity was evaluated across three levels: global strength invariance, network structure invariance (Max *M*), and specific edge invariance. To ensure the robustness of the NCT against the sample size imbalance between groups, a downsampling sensitivity analysis was conducted.

## 3 Results

### 3.1 Demographic and clinical characteristics

A total of 471 adolescents diagnosed with depression were included in this study, comprising 171 patients in the first-episode group and 300 patients in the recurrent group. The sociodemographic and clinical characteristics are summarized in **Table 1**. No significant differences were observed between the two groups regarding gender, educational grade, place of residence, only-child status, school type, family structure, parental education, household income, or digital behavior (all *P* > 0.05). A significant difference was found in the age distribution between the two groups (*P* < 0.001). Consequently, current age was controlled as a covariate in all subsequent network estimations.

**Table 1:** Baseline sociodemographic and clinical characteristics of the first-episode and recurrent groups.

Variables	Total sample (N = 471) n (%)	First-episode (n=171) n (%)	Recurrent (n=300) n (%)	*χ2* value	*P*-value
Age (years)				12.127	<0.001
≤ 15	280 (59.4)	120 (70.2)	160 (53.3)		
16–18	191 (40.6)	51 (29.8)	140 (46.7)		
Gender				2.531	0.112
Male	114 (24.2)	49 (28.7)	65 (21.7)		
Female	357 (75.8)	122 (71.3)	235 (78.3)		
Educational Grade				4.487	0.482
Junior High	253 (53.7)	101 (59.0)	152 (50.6)		
Senior High	218 (46.3)	70 (41.0)	148 (49.4)		
Place of Residence				0.098	0.754
Urban	350 (74.3)	129 (75.4)	221 (73.7)		
Rural	121 (25.7)	42 (24.6)	79 (26.3)		
Only Child				0.101	0.751
Yes	105 (22.3)	40 (23.4)	65 (21.7)		
No	366 (77.7)	131 (76.6)	235 (78.3)		
School Type				1.331	0.249
Key school	180 (38.2)	59 (34.5)	121 (40.3)		
Non-key school	291 (61.8)	112 (65.5)	179 (59.7)		
Family Structure				5.463	0.141
Nuclear family	278 (59.0)	106 (62.0)	172 (57.3)		
Three-generation family	100 (21.2)	30 (17.5)	70 (23.3)		
Extended/Single-parent	93 (19.7)	35 (20.5)	58 (19.3)		
Paternal Education				2.571	0.463
Junior high or below	196 (41.6)	69 (40.3)	127 (42.4)		
Senior high/Vocational	188 (39.9)	64 (37.4)	124 (41.3)		
College degree or above	87 (18.5)	38 (22.2)	49 (16.3)		
Maternal Education				6.452	0.092
Junior high or below	229 (48.6)	75 (43.9)	154 (51.3)		
Senior high/Vocational	168 (35.7)	61 (35.7)	107 (35.7)		
College degree or above	74 (15.7)	35 (20.5)	39 (13.0)		
Per Capita Monthly Income (CNY)				2.367	0.669
≤ 2,999	107 (22.7)	39 (22.8)	68 (22.7)		
3,000 – 4,999	147 (31.2)	55 (32.2)	92 (30.7)		
5,000 – 9,999	134 (28.5)	43 (25.1)	91 (30.3)		
≥ 10,000	83 (17.6)	34 (19.9)	49 (16.3)		
Daily Internet Use				3.92	0.27
≤ 3 hours	123 (26.1)	48 (28.1)	75 (25.0)		
3–5 hours	109 (23.1)	46 (26.9)	63 (21.0)		
> 5 hours	239 (50.7)	77 (45.0)	162 (54.0)		
Years of Using Electronic Devices				1.111	0.775
≤ 5 years	300 (63.7)	112 (65.5)	188 (62.7)		
5–10 years	149 (31.6)	50 (29.2)	99 (33.0)		
> 10 years	22 (4.7)	9 (5.3)	13 (4.3)		
Age of First Onset				1.194	0.275
0–12 years	151 (32.1)	49 (28.7)	102 (34.0)		
13–18 years	320 (67.9)	122 (71.3)	198 (66.0)		
Family History of Mental Illness				0.001	0.975
Yes	54 (11.5)	19 (11.1)	35 (11.7)		
No	417 (88.5)	152 (88.9)	265 (88.3)		
Physical Comorbidities				0.033	0.857
Yes	33 (7.0)	11 (6.4)	22 (7.3)		
No	438 (93.0)	160 (93.6)	278 (92.7)		
History of Suicidal Behavior				0.014	0.905
Yes	306 (65.0)	110 (64.3)	196 (65.3)		
No	165 (35.0)	61 (35.7)	104 (34.7)		

Data are presented as n (%). *P* values were calculated using the Pearson chi-square test. Certain subcategories with sparse sample sizes were rationally merged to meet the assumptions for valid statistical testing (e.g., extended families and single-parent families were merged into a single category for analysis).

### 3.2 Descriptive statistics and overall network structure

#### 3.2.1 Descriptive statistics of study variables

**Table 2** presents the mean scores and standard deviations for the 14 study dimensions encompassing internet social support, school connection, social adaptation, family care, self-esteem, and depressive symptoms. No extreme floor or ceiling effects appeared in the scale scores across the total sample or the clinical subgroups.

**Table 2:** Descriptive statistics of the 14 dimensions (N = 471).

Dimension	Total (N = 471) mean ± SD	First-episode (n=171) mean ± SD	Recurrent (n=300) mean ± SD	Predictability (*R*^2^)	Bridge expected influence	Node number
Internet social support						
Information Support	18.68 ± 3.92	18.49 ± 3.87	18.80 ± 3.95	0.170	0.470	Node 1
Peer Support	26.51 ± 9.25	25.99 ± 9.35	26.80 ± 9.20	0.372	-0.035	Node 2
Emotional Support	14.38 ± 4.99	14.16 ± 4.60	14.51 ± 5.21	0.528	0.115	Node 3
Instrumental Support	12.76 ± 4.88	12.67 ± 4.79	12.82 ± 4.94	0.436	0.174	Node 4
School connection						
Classmate Support	10.06 ± 2.94	10.03 ± 3.04	10.08 ± 2.88	0.428	0.228	Node 5
Teacher Support	8.85 ± 2.04	8.82 ± 1.98	8.86 ± 2.08	0.235	0.126	Node 6
School Belonging	6.58 ± 2.86	6.74 ± 2.93	6.49 ± 2.82	0.486	0.209	Node 7
Social adaptation						
Interpersonal Harmony	37.99 ± 12.38	39.21 ± 12.62	37.30 ± 12.21	0.564	0.658	Node 8
Environment Identity	21.39 ± 5.13	21.64 ± 5.08	21.25 ± 5.17	0.455	0.071	Node 9
Life Independence	11.88 ± 3.47	11.99 ± 3.34	11.82 ± 3.55	0.301	0.103	Node 10
Learning Autonomy	16.67 ± 5.51	17.33 ± 5.02	16.30 ± 5.74	0.433	0.163	Node 11
Core psychological factors						
Family Care Index	3.10 ± 2.79	3.17 ± 2.79	3.06 ± 2.79	0.158	0.417	Node 12
Self-Esteem	20.47 ± 5.54	20.21 ± 5.62	20.62 ± 5.49	0.450	0.033	Node 13
Depressive Symptoms	19.57 ± 8.00	19.50 ± 7.85	19.60 ± 8.10	0.367	-0.721	Node 14

SD, Standard Deviation. Predictability (*R*^2^) represents the proportion of variance of a specific node that can be explained by all its neighboring nodes within the overall network, calculated using the *mgm* package. Bridge Expected Influence (Bridge EI) quantifies the net sum (algebraic sum) of the weights of all edges connecting a specific node to nodes in other predefined measurement communities, accounting for both positive and negative associations (calculated via the *networktools* package). Higher Bridge EI indicates a stronger cumulative magnitude of positive or negative partial correlations with other psychological sub-systems, while a Bridge EI near zero suggests a neutralization of cross-community influences.

#### 3.2.2 Overall network topology and centrality

**Figure 1:**
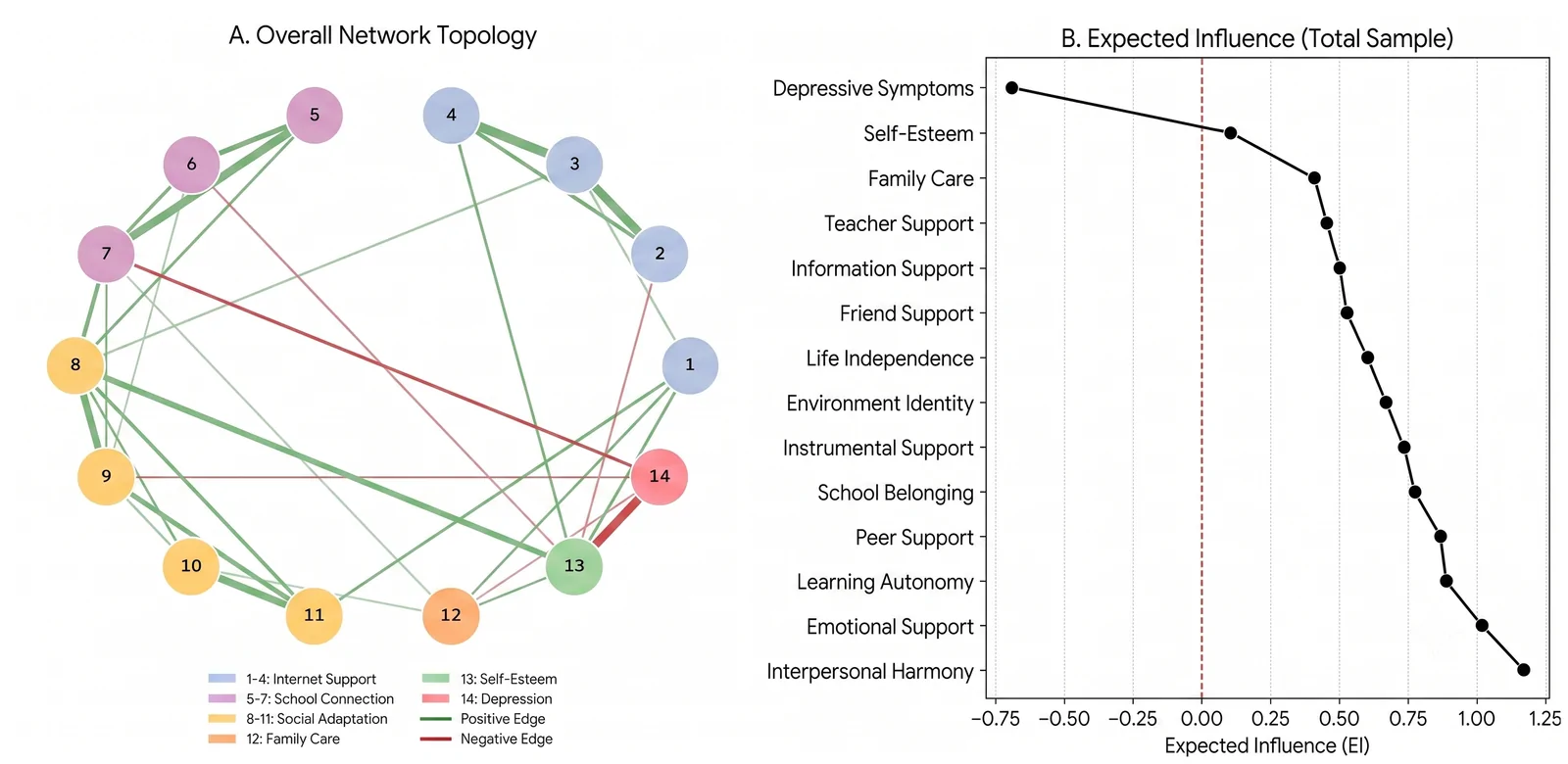
Overall psychosocial ecosystem network and node centrality. **(A)** The network topology of the 14 psychosocial dimensions in the total sample (N = 471). Green and red edges represent positive and negative partial correlations, respectively. Edge thickness indicates the strength of the association. **(B)** Expected Influence (EI) of each node within the overall network, illustrating the net associative strength. interpersonal harmony exhibits the highest positive influence, while depressive symptoms exhibit the strongest negative influence.

An EBICglasso network model was estimated for the total sample (**Figure 1A**). Visual inspection and the edge weight matrix indicated that the strongest negative partial correlation was observed between Self-Esteem and Depressive Symptoms. Regarding node centrality (**Figure 1B**), Interpersonal Harmony exhibited the highest Expected Influence, identifying it as the primary central hub in the psychosocial network. Meanwhile, Self-Esteem exhibited a unique topological profile: although it maintained extensive cross-community connections, its Bridge Expected Influence (Bridge EI) was near zero (0.033), indicating a neutralization of positive and negative bridging effects (**Table 2**). Furthermore, node predictability (*R*^2^) was calculated to quantify the variance of each node explained by its neighboring nodes. Interpersonal Harmony (*R*^2^ = 0.564) and Emotional Support (*R*^2^ = 0.528) showed the highest predictability.

Network stability analyses indicated that the CS-coefficient for Expected Influence was 0.75, exceeding the recommended threshold of 0.5. The nonparametric bootstrap analysis revealed stable 95% confidence intervals for the edge weights (**Supplementary Materials** **Supplementary Figures 1**, **2**).

#### 3.2.3 Bridge centrality analysis

Bridge Expected Influence was calculated to identify nodes connecting the predefined psychosocial sub-systems (i.e., Internet Support, School Connection, Social Adaptation, Family Care, Core Psychological factors, and Depression), as detailed in **Table 2**. Self-Esteem and Depressive Symptoms exhibited the most significant bridge positions. Notably, Depressive Symptoms showed the strongest negative bridge influence (Bridge EI = -0.721), while Self-Esteem showed a neutralized bridge influence (Bridge EI = 0.033). Among environmental and social support variables, Interpersonal Harmony (Bridge EI = 0.658) and Family Care Index (Bridge EI = 0.417) emerged as the most influential positive bridges connecting different measurement communities.

### 3.3 Network comparison between first-episode and recurrent groups

**Figure 2:**
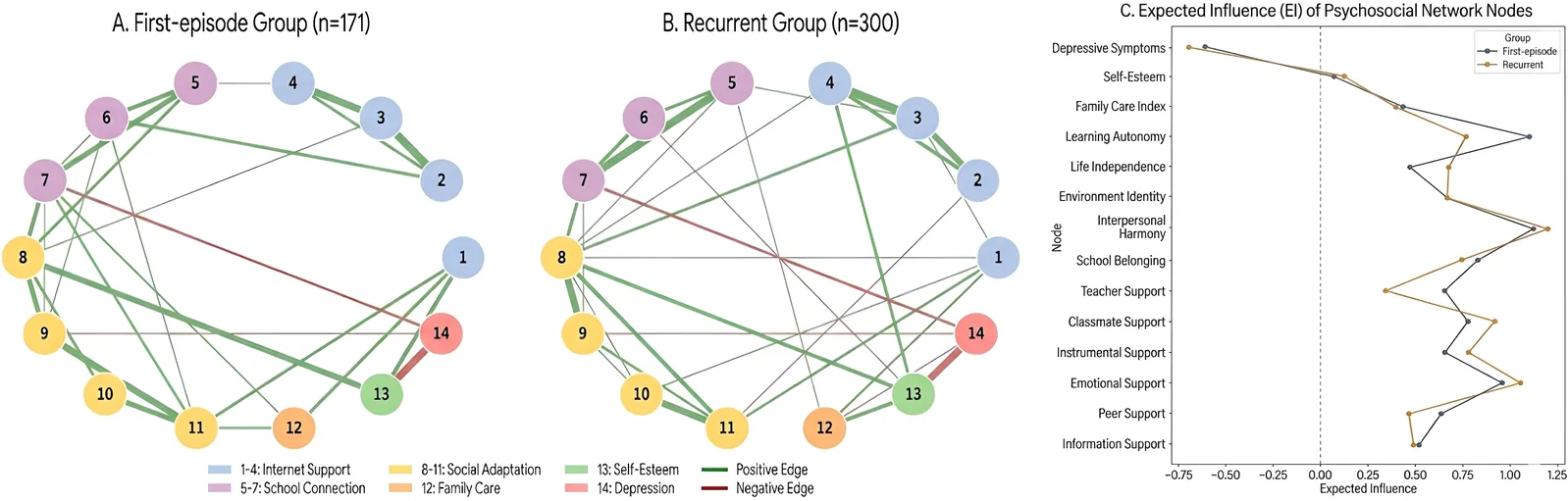
Structural comparison of psychosocial networks between clinical stages. **(A)** The network topology for the first-episode group (n=171). **(B)** The network topology for the recurrent group (n=300). Both networks were estimated using Gaussian Graphical Models with EBICglasso regularization. **(C)** Comparison of Expected Influence (EI) between the two clinical stages. The line chart illustrates changes in the relative importance and overall expected influence of nodes as the disease progresses to the recurrent stage.

Networks for the first-episode and recurrent groups were estimated separately (**Figure 2**). A Network Comparison Test (NCT) with 1000 permutations was conducted to statistically evaluate the differences between the two networks (**Table 3**; Part A). The global strength invariance test showed no significant difference between the two networks (*S* = 0.152, *P* = 0.212). However, the network structure invariance test indicated a statistically significant difference in the maximum edge weight (*M* = 0.243, *P* = 0.039). Furthermore, a downsampling sensitivity analysis confirmed that the core specific edge differences and overall topological heterogeneity remained robust, despite the expected loss of statistical power (see **Supplementary Table 1**).

**Table 3:** Results of the network comparison test between first-episode and recurrent groups.

Part A: global network metrics				
Metric	Test Statistic			*P*-value
Global Strength Invariance	S=0.152			0.212
Network Structure Invariance (Max Edge)	M=0.243			0.039*
Part B: significant specific edge differences				
Edge (Node A ↔ Node B)	First-episode	Recurrent	Difference	P-value
Weakened connections in the recurrent group				
Environment Identity (9) ↔ Learning Autonomy (11)	0.353	0.126	0.227	< 0.01**
Family Care Index (12) ↔ Learning Autonomy (11)	0.138	0	0.138	< 0.01**
School Belonging (7) ↔ Learning Autonomy (11)	0.113	0	0.113	< 0.05*
Strengthened connections in the recurrent group				
Family Care Index (12) ↔ Self-Esteem (13)	0	0.187	-0.187	< 0.01**
Interpersonal Harmony (8) ↔ Learning Autonomy (11)	0.062	0.207	-0.145	< 0.05*
Information Support (1) ↔ Life Independence (10)	0	0.081	-0.081	< 0.05*
Emotional Support (3) ↔ Classmate Support (5)	0	0.075	-0.075	< 0.05*

NCT was performed using 1000 permutations, with age adjusted as a covariate. In Part B, edge weights represent the regularized partial correlations. A positive difference indicates a stronger connection in the first-episode group (collapse in recurrence), while a negative difference indicates a stronger connection in the recurrent group (compensation). *P < 0.05, **P < 0.01, ***P < 0.001.

### 3.4 Specific edge differences between the first-episode and recurrent groups

To evaluate the specific topological differences between the two clinical stages, specific edge invariance was analyzed using the EBICglasso algorithm. **Table 3** (Part B) details the significant specific edge-weight differences between the networks.

In the recurrent group, several edges exhibited significantly lower weights compared to the first-episode group. Specifically, the partial correlation between Family Care Index (Node 12) and Learning Autonomy (Node 11) was present in the first-episode group (0.138) but absent (0.000) in the recurrent group (Difference = 0.138, *P* < 0.01). Similarly, the edge connecting School Belonging (Node 7) and Learning Autonomy (Node 11) shrank to zero in the recurrent group (Difference = 0.113, *P* < 0.05). Additionally, the association between Environment Identity (Node 9) and Learning Autonomy (Node 11) was significantly lower in the recurrent group compared to the first-episode group (Difference = 0.227, *P* < 0.01).

Conversely, certain edges demonstrated significantly higher weights in the recurrent group. The association between Family Care Index (Node 12) and Self-Esteem (Node 13) was absent in the first-episode group but emerged in the recurrent group (0.187, Difference = -0.187, *P* < 0.01). The partial correlation between Interpersonal Harmony (Node 8) and Learning Autonomy (Node 11) was also significantly stronger in the recurrent network (Difference = -0.145, *P* < 0.05). Furthermore, new edges related to digital internet support were present in the recurrent group but absent in the first-episode group, including the associations between Information Support (Node 1) and Life Independence (Node 10) (Difference = -0.081, *P* < 0.05), and between Emotional Support (Node 3) and Classmate Support (Node 5) (Difference = -0.075, *P* < 0.05).

## 4 Discussion

Through network analysis, this study reveals the structural features of psychosocial support for depressed adolescents, and the structural heterogeneity between the first-episode and recurrent group as well.

The results indicate that interpersonal harmony (Node 8) functions as both a core node and a key bridge node in the psychosocial network of adolescents with depression, demonstrating the highest expected influence (EI = 1.170) and bridge expected influence (Bridge EI = 0.658). As the central processing hub of the network, interpersonal harmony (Node 8) exhibits extensive connections across different types of resources. Consistent with ecological systems theory (16), this centrality indicates that objective external resources, such as family care (Node 12) and school belonging (Node 7), are closely linked to positive interpersonal experiences, which further correlate with internal protective factors like self-esteem (Node 13). Furthermore, according to interpersonal theory, adolescence is a critical phase where emotional stability and psychological defenses rely heavily on peer acceptance and social feedback. Interpersonal harmony (Node 8) represents the actual success of social adaptation and serves as the central processing hub of real-world social functioning. Maintaining positive communication and experiencing fewer interpersonal conflicts are directly associated with better environmental support and lower depressive symptoms. Empirical studies consistently indicate that interpersonal interaction factors are closely linked to depressive symptom severity (Node 14). Specifically, positive peer relationships (Node 2) are associated with lower symptom severity (31), whereas communication skills and interpersonal conflict show strong correlations with depressive symptom levels (32, 33). Therefore, clinical nursing interventions for depressed adolescents should extend beyond basic symptom control. Prioritizing the improvement of interpersonal difficulties as a primary intervention target is recommended, as this core node exhibits strong partial correlations with broader positive adaptations across the entire psychosocial system. More importantly, its high bridge expected influence (Bridge EI = 0.658) explains how online social support connects to real-world psychological health. When depressed adolescents experience social withdrawal offline, they frequently turn to the internet for emotional or peer support (Nodes 1–4). However, our network topology clearly shows that these online support dimensions do not have strong, direct bridging connections to depressive symptoms (Node 14). Instead, interpersonal harmony (Node 8) acts as the connecting bridge. For online emotional support (Node 3) or peer support (Node 2) to correlate with lower real-world symptom severity, adolescents need to apply the social confidence or communication skills practiced online to improve their actual face-to-face interpersonal relationships (Node 8). This bridging role explains how digital help-seeking connects to real-world recovery factors like self-esteem (Node 13) and family care (Node 12).

The topological profile of self-esteem (Node 13) in the overall network requires specific attention with a near-zero bridge expected influence (Bridge EI = 0.033) which indicates self-esteem works as a system stabilizer rather than a lack of importance (34). It reflects the algebraic neutralization of strong positive and negative associations (28). Healthy self-esteem reflects how individuals process negative information, when facing academic setbacks or social conflicts, adolescents with higher self-esteem are less likely to blame themselves. This cognitive pattern of avoiding excessive self-blame is closely associated with a lower risk of depression (35).Conversely, many studies show that the persistent self-denial linked to low self-esteem is strongly associated with more severe depressive symptoms (36).Also, a stable self-esteem system relates to better psychological resilience and lower emotional distress in depressed adolescents (35). Thus, the near-zero bridge expected influence of self-esteem in our network does not signify irrelevance but rather a balancing function. Self-esteem (Node 13) exhibits positive partial correlations that algebraically offset its negative association with depressive symptoms (Node 14) while integrating positive external support. Since self-esteem serves as a “system stabilizer” in the overall network of adolescents with depression, the future clinical interventions should shift from simply alleviating depressive symptoms or merely enhancing self-esteem to training the cognitive rebalancing function of self-esteem. Specific measures could be like internalizing positive external support, learning to avoid immediate self-blame when encountering negative feedback, monitoring the dynamic association between self-esteem and symptoms rather than their absolute levels, et al.

The Network Comparison Test confirmed significant structural heterogeneity between the first episode and recurrent group (M = 0.243, *P* = 0.039). This finding aligns with previous psychiatric studies demonstrating that neural network connectivity and reward processing differ at different stage of depression (37, 38). One aspect of the structural heterogeneity lies in the shifting connections of family care (Node 12). Specifically, while family care (Node 12) and school belonging (Node 7) were positively correlated with learning autonomy (Node 11) in the first-episode network, these associations were absent in the recurrent stage (P < 0.05), where family care (Node 12) instead formed a close and specific link with the patient’s self-esteem (Node 13). Such a shift probably reflects a fundamental change in family’s understanding of the disease. Using a life course perspective, Wang and colleagues conducted in-depth qualitative interviews with 17 caregivers of adolescents with bipolar disorder. The results revealed that in the early stage of the illness, caregivers generally exhibited denial and avoidance regarding the disorder, attributing the adolescents’ behaviors to excessive academic pressure, they concerned more about their children’s school belongings and feelings towards study. However, as caregiving progressed, their understanding of the illness deepened, they came to recognize the importance of teaching the child to live with the illness, with future expectations shifting toward respecting the child’s own wishes (39). Although depression and bipolar disorder are distinct conditions, the caregiving experiences may share considerable similarities. A study analyzing the caregiving burden among 300 caregivers of patients with mental disorders (including both depression and bipolar disorder) found no significant difference in caregiving burden across different diagnoses (40). Therefore, the findings from Wang et al. may, to some extent, reflect the caregiving trajectory of caregivers of adolescents with depression. These findings have two practical implications. At the public health level, efforts should be made to improve mental health literacy and reduce stigma, thereby enabling earlier detection and timely treatment of adolescent mood disorders. At the clinical level, once a diagnosis is established, family-based support should be initiated promptly rather than focusing solely on the patient, given that family care is strongly linked to recovery outcomes.

Another aspect of structure heterogeneity relates to digital network compensation. In the recurrent group, real-world support connections were weaker, while associations with online social support were more prominent. Compared with network of first-episode group, the network of recurrent group is much more complicated, but only two connections are statistically significant. One connection shows that a positive link exists between online information support (Node 1) and patients’ life independence (Node 10). This finding is consistent with Croke et al (41), who reported that digital interventions involving informational exchange were associated with moderate improvements in patient activation (SMD = 0.39, 95% CI 0.23–0.55). Patient activation includes the knowledge and skills to manage one’s own health, translating into independent living behaviors such as self-medication management, early recognition of relapse signs, and proactive help-seeking. Another connection demonstrated that online emotional support (Node 3) was also positively associated with classmate support (Node 5). Smit et al (42) provided evidence from a longitudinal study of 301 patients with depression. In that study, patients used an online peer support community called Depression Connect. The results showed that participation in the online community was associated with higher levels of belonging, self-efficacy, and empowerment. Additionally, qualitative findings suggested that online interactions served as a “practice ground for self-disclosure” in the offline world. Discussing social interactions and behavioral patterns with peers in the community was associated with the rebuilding of social confidence and with connections to real world relationships. Online emotional support (Node 3) was not a replacement for offline peer support (Node 2); instead, both coexisted. Therefore, in clinical practice, for recurrent patients, nurses may provide standardized online information modules to support self-management during follow up care. They may also use online emotional support communities as a transition for social skills practice. At the same time, nurses can provide digital health literacy education, including information verification and screen time control.

### 4.1 Clinical implications

Based on the topological characteristics and structural heterogeneity revealed in this cross-sectional network analysis, we propose both overarching and clinical-stage-specific hypotheses for psychiatric nursing assessments and potential intervention targets in adolescent depression. It must be noted that because Gaussian Graphical Models exclusively model contemporaneous partial correlations, the following clinical pathways represent correlational associations that warrant validation through longitudinal and interventional trial designs.

At the macro level across all clinical stages, nursing assessments and intervention models should align with the global topology of the psychosocial ecosystem. Because Interpersonal Harmony (Node 8) and Self-Esteem (Node 13) emerge as the primary central hub and system stabilizer, respectively, addressing relational difficulties represents a high-priority clinical candidate strongly associated with broader psychosocial well-being. Rather than focusing solely on basic symptom management, nurses should prioritize interpersonal functioning, as enhancements in this central hub are robustly correlated with positive adaptations across other psychosocial dimensions. Furthermore, clinical strategies incorporating cognitive rebalancing training for self-esteem—guiding adolescents to process negative feedback and setbacks without immediate self-blame—may be particularly relevant, given its strong inverse association with Depressive Symptoms (Node 14).

At the stage-specific level, the observed structural heterogeneity highlights distinct clinical pathways tailored to different phases of the illness: For first-episode patients, robust positive associations remain intact among family care (Node 12), school belonging (Node 7), and learning autonomy (Node 11). Nursing interventions should focus on reinforcing these intact environmental resources. School and community nurses could vigorously promote Family-School Collaborative Interventions, leveraging these strongly correlated network edges as structural buffers associated with the maintenance of a stable equilibrium between environmental support and academic autonomy (Learning Autonomy, Node 11).

For recurrent patients, the network topology reveals a decoupling of academic adaptation from traditional real-world support systems, accompanied by strengthened specific associations in family and digital domains. First, because the association between academic adaptation and external support is absent in this stage, interventions demanding high academic expectations may be less clinically relevant. Instead, since Family Care Index (Node 12) forms a specific, prominent link with Self-Esteem (Node 13) in recurrent networks, nursing support should emphasize unconditional emotional companionship. Clinicians should guide exhausted caregivers to shift their focus from academic supervision to serving as “self-worth guardians,” a role closely linked to stabilizing the fragile self-esteem of adolescents experiencing recurrent episodes. Moreover, family-based support should be initiated promptly to help caregivers adapt to the prolonged caregiving trajectory.

Finally, given the prominent digital compensation correlations observed in recurrent patients, interventions should explore structured digital empowerment strategies. To address poor self-medication management and relapse recognition, nurses could integrate standardized online information modules—such as relapse checklists and medication reminders—into discharge education, as online information support (Node 1) is positively associated with Life Independence (Node 10). Concurrently, online emotional mutual-support communities could be utilized as safe, low-risk intermediate environments associated with enhanced social self-efficacy and peer belonging. By safely practicing self-disclosure online, patients may gradually rebuild interpersonal confidence, which may serve as an intermediate step associated with improved real-world interactions (Classmate Support, Node 5). Despite these potential benefits, nurses must remain vigilant regarding unreliable online information and internet overreliance by incorporating digital health literacy modules into follow-up care, emphasizing information verification and balanced online-offline usage.

### 4.2 Limitations

This study presents several limitations. Firstly, the cross-sectional design only reveals statistical associations and precludes the establishment of strict causal relationships between network nodes. Future studies should employ longitudinal tracking designs to observe the dynamic evolution of these connections over time. Secondly, the sample was drawn exclusively from two tertiary hospitals in Changsha, Hunan Province, which may restrict the external validity and generalizability of the findings to adolescents in different cultural or healthcare contexts. Finally, the current network model did not incorporate detailed clinical confounders such as specific psychiatric medication regimens. Given that psychotropic medications may influence cognitive drive and learning autonomy, future studies should control for pharmacotherapy variables to validate the observed decoupling of academic adaptation.

## 5 Conclusion

In summary, this study utilized network analysis to reveal significant structural heterogeneity in the psychosocial ecosystems of adolescents across clinical stages of depression. Globally, interpersonal harmony exhibits the highest activating centrality as the primary hub and stabilized by self-esteem. However, as the disease becomes recurrent, the structural focus fundamentally shifts: real-world academic associations disappear, while family care forms a specific correlation with self-esteem regulation and digital platforms provide compensatory social connections. These findings emphasize that psychiatric nursing cannot rely on static strategies. Interventions must universally target interpersonal relationships and the cognitive rebalancing of self-esteem. Moreover, stage-specific care for recurrent patients must urgently prioritize family-based self-esteem regulation and the strategic use of digital communities as transitional bridges for social adaptation.

## Data Availability

The raw data supporting the conclusions of this article will be made available by the authors, without undue reservation.
