# Supplementary material for: Structural heterogeneity of the psychosocial network in adolescent depression: a comparison between first-episode and recurrent patients
Source: Front Psychiatry. 2026 Aug 13;17:1918132. doi: 10.3389/fpsyt.2026.1918132 (PMC13519595; doi:10.3389/fpsyt.2026.1918132)
Supplement: Supplementary file 1 [file Image1.pdf]

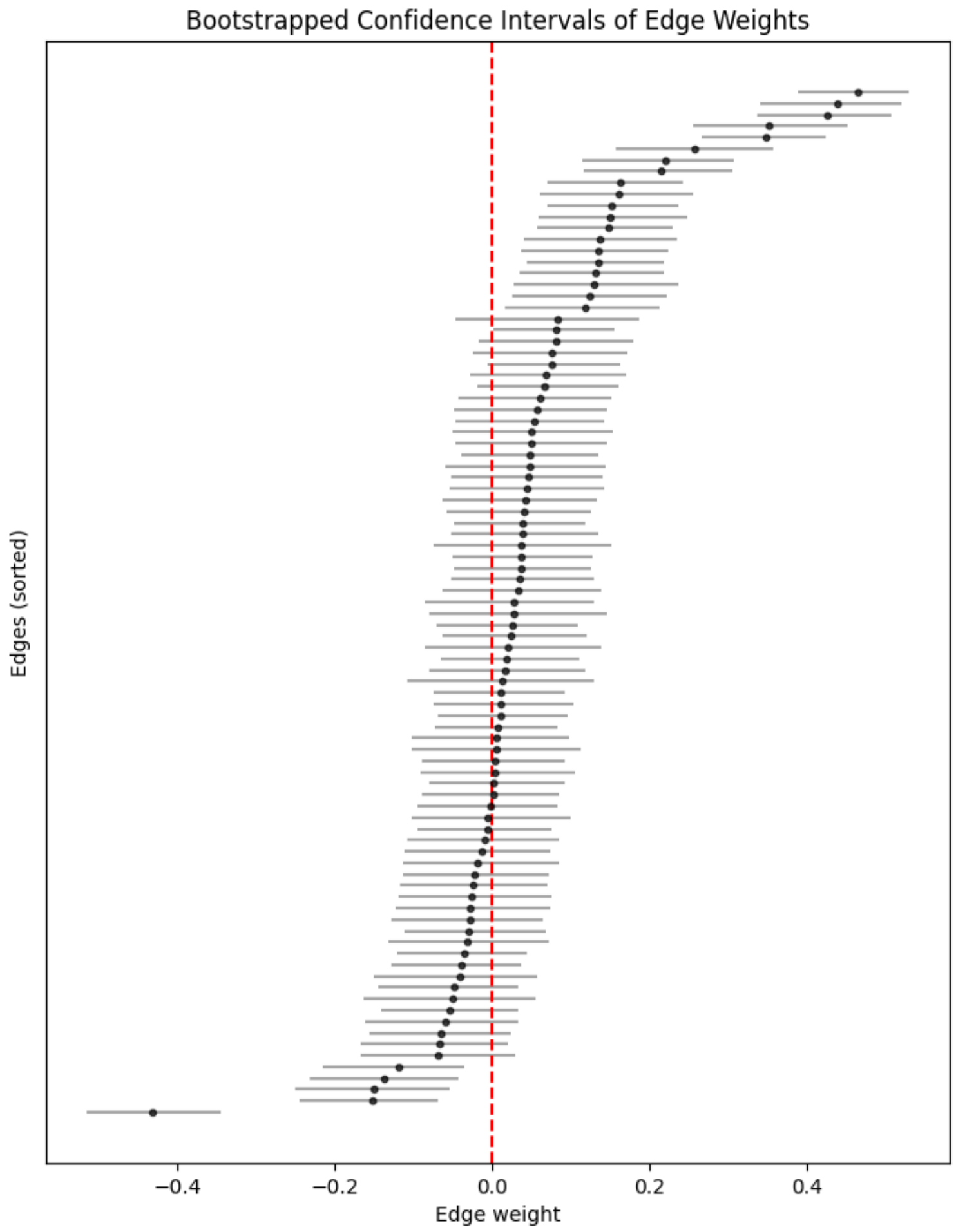

Supplementary Figure S1. Bootstrapped 95% confidence intervals of estimated edge weights for the overall psychosocial network. The red line indicates the estimated sample values of the edge weights, and the gray area represents the bootstrapped 95% confidence intervals (CIs) based on 1,000 nonparametric bootstrap iterations. Each horizontal line represents one specific edge in the network, ordered from the highest positive edge weight to the lowest negative edge weight. The relatively narrow CIs indicate that the accuracy of the edge weight estimation in the current Gaussian Graphical Model is highly reliable.

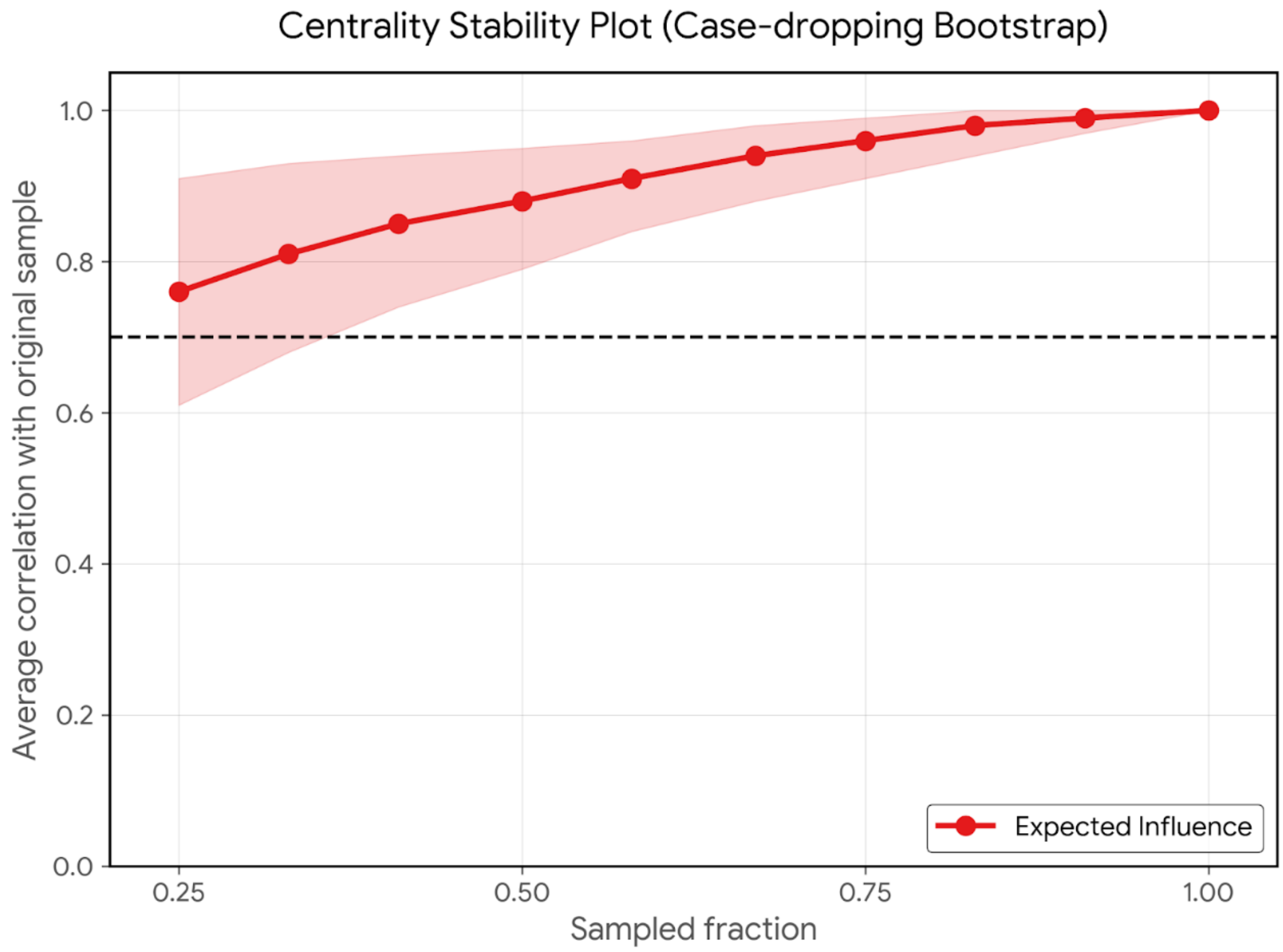

Supplementary Figure S2. Centrality stability plot of Expected Influence (EI) for the overall psychosocial network. This plot illustrates the stability of the Expected Influence centrality index under a case-dropping bootstrap procedure (1,000 iterations). The x-axis represents the proportion of cases retained from the original sample, and the y-axis indicates the average correlation between the centrality indices of the original network and those of the bootstrapped subsamples. The red area reflects the 95% confidence intervals. The Correlation Stability coefficient (CS-coefficient) for Expected Influence was 0.75, which significantly exceeds the recommended strict threshold of 0.50, demonstrating that the node centrality estimations are highly robust.
