# Supplementary material for: Structural heterogeneity of the psychosocial network in adolescent depression: a comparison between first-episode and recurrent patients
Source: Front Psychiatry. 2026 Aug 13;17:1918132. doi: 10.3389/fpsyt.2026.1918132 (PMC13519595; doi:10.3389/fpsyt.2026.1918132)
Supplement: Supplementary file 3 [file Table1.docx]

| Metric / Specific Edge | Primary Analysis (n=171 vs. 300) | Sensitivity Analysis (n=171 vs. 171) |  |
| --- | --- | --- | --- |
| Global Network Metrics | Test Statistic (*P*-value) | Mean Test Statistic (Mean *P*-value) |  |
| Global Strength Invariance (S) | S = 0.152 (*P* = 0.212) | S = 0.168 (*P* = 0.285) |  |
| Network Structure Invariance (Max M) | M = 0.243 (*P* = 0.039*) | M = 0.262 (*P* = 0.065) |  |
| Weakened Connections (Recurrent) | Difference (*P*-value) | Mean Difference (Mean P-value) |  |
| Environment Identity (9) ↔ Learning Autonomy (11) | 0.227 (*P* < 0.01**) | 0.231 (*P* = 0.008**) |  |
| Family Care Index (12) ↔ Learning Autonomy (11) | 0.138 (*P* < 0.01**) | 0.142 (*P* = 0.018*) |  |
| School Belonging (7) ↔ Learning Autonomy (11) | 0.113 (*P* < 0.05*) | 0.118 (*P* = 0.038*) |  |
| Strengthened Connections (Recurrent) | Difference (*P*-value) | Mean Difference (Mean *P*-value) |  |
| Family Care Index (12) ↔ Self-Esteem (13) | -0.187 (*P* < 0.01**) | -0.195 (*P* = 0.012*) |  |
| Interpersonal Harmony (8) ↔ Learning Autonomy (11) | -0.145 (*P* < 0.05*) | -0.149 (*P* = 0.041*) |  |
| Information Support (1) ↔ Life Independence (10) | -0.081 (*P* < 0.05*) | -0.085 (*P* = 0.048*) |  |
| Emotional Support (3) ↔ Classmate Support (5) | -0.075 (*P* < 0.05*) | -0.078 (*P* = 0.052) |  |

**Supplementary Table S1. Results of the Downsampling Bootstrap Sensitivity Analysis for Network Comparison Between the First-Episode (**$\boldsymbol{n=171}$**) and Recurrent (**$\boldsymbol{n=171}$**) Groups**

**Note:** The sensitivity analysis was performed by randomly downsampling the recurrent grou*p* to match the sample size of the first-episode grou*p* (n=171) across multiple iterations. Results reflect the mean test statistics and P-values from the downsampled permutations. While the global structure invariance (Max M) shifted to marginal significance due to the reduced statistical power (N decreased from 471 to 342), the effect sizes (absolute differences) and the specific core edge differences remained highly stable and predominantly significant, confirming the robustness of the structural heterogeneity. **P* < 0.05, *P* < 0.01.
